# Transmission and Selection of Macrolide Resistant *Mycoplasma genitalium* Infections Detected by Rapid High Resolution Melt Analysis

**DOI:** 10.1371/journal.pone.0035593

**Published:** 2012-04-20

**Authors:** Jimmy Twin, Jorgen S. Jensen, Catriona S. Bradshaw, Suzanne M. Garland, Christopher K. Fairley, Lim Yi Min, Sepehr N. Tabrizi

**Affiliations:** 1 Department of Microbiology and Infectious Diseases, The Royal Women's Hospital, Melbourne, Australia; 2 Murdoch Childrens Research Institute, Melbourne, Australia; 3 Statens Serum Institut, Copenhagen, Denmark; 4 Melbourne Sexual Health Centre, The Alfred Hospital, Melbourne, Australia; 5 Department of Epidemiology and Preventive Medicine, Monash University, Melbourne, Australia; 6 Melbourne School of Population Health, University of Melbourne, Melbourne, Australia; 7 Department of Obstetrics and Gynecology, University of Melbourne, Melbourne, Australia; 8 Department of Microbiology, The Royal Children's Hospital, Melbourne, Australia; Auburn University, United States of America

## Abstract

**Background:**

*Mycoplasma genitalium* (MG) causes urethritis, cervicitis and pelvic inflammatory disease. The MG treatment failure rate using 1 g azithromycin at an Australian Sexual Health clinic in 2007–9 was 31% (95%CI 23–40%). We developed a rapid high resolution melt analysis (HRMA) assay targeting resistance mutations in the MG 23S rRNA gene, and validated it against DNA sequencing by examining pre- and post-treatment archived samples from MG-infected patients.

**Methodology/Principal Findings:**

Available MG-positive pre-treatment (n = 82) and post-treatment samples from individuals with clinical treatment failure (n = 20) were screened for 23S rRNA gene mutations. Sixteen (20%) pre-treatment samples possessed resistance mutations (A2058G, A2059G, A2059C), which were significantly more common in patients with symptomatic azithromycin-treatment failure (12/26; 44%) than in those clinically cured (4/56; 7%), p<0.001. All 20 patients experiencing azithromycin-failure had detectable mutations in their post-treatment samples. In 9 of these cases, the same mutational types were present in both pre- and post-treatment samples indicating transmitted resistance, whilst in 11 of these cases (55%), mutations were absent in pre-treatment samples indicating likely selection of resistant isolates have occurred. HRMA was able to detect all mutational changes determined in this study by DNA sequencing. An additional HRMA assay incorporating an unlabelled probe was also developed to detect type 4 single-nucleotide polymorphisms found in other populations, with a slightly lower sensitivity of 90%.

**Conclusions/Significance:**

Treatment failure is associated with the detection of macrolide resistance mutations, which appear to be almost equally due to selection of resistant isolates following exposure to 1 g azithromycin and pre-existing transmitted resistance. The application of a rapid molecular assay to detect resistance at the time of initial detection of infection allows clinicians to shorten the time to initiate effective second line treatment. This has the potential to reduce transmission of resistant strains and to avoid sequelae associated with persistent untreated infection.

## Introduction

*Mycoplasma genitalium* was first isolated in 1980 from men with non-gonococcal urethritis (NGU) [1] [2] and has since been established as a sexually transmitted infection [3] responsible for 20–35% of non-chlamydial NGU, as well as cervicitis. Evidence is mounting to suggest that MG plays a role in other conditions such as endometritis, pelvic inflammatory disease and tubal factor infertility in women [4], [5], [6], [7], [8] plus balanoposthitis in men [9]. As MG is a fastidious organism, making a diagnosis with conventional culture technique is difficult and requires the use of sensitive nucleic acid amplification tests (NAATs). Commercially available kits are becoming available for the detection of MG, such as the AmpliSens® *Mycoplasma genitalium*-EPh PCR kit, Bio-Rad Dx CT/NG/MG assay, EuroClone DUPLICα kit, and Gen-Probe; however none as yet have obtained US FDA approval. Hence current testing is predominately based on clinical services with validated in-house PCR assays [10]. To accurately determine whether an MG infection has been successfully treated, a test of cure (TOC) using PCR-based assays is generally deferred for at least 2–4 weeks, to avoid the detection of non-viable DNA that can persist post-treatment resulting in false positive results [11]. This limitation results in significant delays in establishing whether an infection has been adequately eradicated, with the potential for ongoing transmission and persistent symptoms for infected individuals.

The current recommended treatment for MG infection is the macrolide antibiotic azithromycin [12]. One comparative trial and one observational study have both shown that a single 1 g dose of azithromycin is significantly more effective than the multi-dose doxycycline, with cure rates of 86–87% compared with 22–45% respectively [11], [13]. In addition an extended azithromycin regimen (500 mg on day 1 followed by 250 mg daily for 4 days) cured >95% of doxycycline treatment failure cases. The extended regimen of azithromycin has been evaluated in a number of observational studies with cure rates reported to range from 74–96% [11], [14].

Routine testing for MG commenced at the Melbourne Sexual Health Centre (MSHC), Australia, in 2005 for symptomatic individuals presenting with urethritis, cervicitis, pelvic inflammatory disease or of sexual contacts of MG-infected individuals. Two clinical audits (March 2005 to November 2007 and December 2007 to August 2009) were conducted following the onset of testing to determine the effectiveness of 1 g azithromycin for treating MG infection. The efficacy of azithromycin was found to be 84% in the first audit period [15], and declined to 69% in the second audit period (p<0.001) [16]. Previous work had identified a number of macrolide resistance mutations (A2058G, A2059G) in a small subset of individuals experiencing treatment failure with azithromycin at MSHC in 2004. However, the proportion of cases with treatment failure attributable to azithromycin resistance mutations in the clinical population and how many were likely due to transmitted (pre-existing) resistance compared to those selected for (following azithromcyin) was not known [17].

The mode of action of macrolide antibiotics such as azithromycin is preventing bacterial multiplication by binding to the 50S subunit of the ribosome (which includes the 5S and 23S subunits) and inhibiting translation of mRNA; hence interfering with protein synthesis. Resistance to macrolide antibiotics can occur through mutational changes that in turn inhibit binding of the macrolide. Such mutational changes, in particular those occurring in nucleotide positions 2058 and 2059 in region V of the 23S rRNA gene (*E. coli* numbering), have been well characterized in those of the bacterial class Mollicutes such as *M. pneumoniae*, a close relative of MG [18]. The macrolide resistance seen in *M. pneumoniae* infections is increasing at an alarming rate particularly in Asia, among patients with community-acquired pneumonia and respiratory tract infections, primarily as the result of an increase in circulation of these resistant strains in the community [19], [20]. This resistance pattern has since been documented with MG infections, being predominantly adenine to guanine transitions in either 23S rRNA gene position 2058 or 2059 [17], [21], [22], [23].

Rapid assays have been developed for the detection of macrolide resistance in *M. pneumonia*e based on High Resolution Melt Analysis (HRMA) [24], [25]. HRMA is performed when double-stranded DNA is exposed to increasing temperature gradient for gradual denaturing from double-stranded to single-stranded DNA, at a rate based on its nucleic acid content. Different nucleic acid compositions will therefore denature at a different rate and HRMA is ideally able to discriminate a single base change in the target DNA. The incorporation of a sensitive fluorescent dye that intercalates only with double-stranded DNA allows for the real-time monitoring of the melt profiles, giving a measureable profile that can be compared to standards. HRMA is a relatively inexpensive and rapid molecular method for the detection of single nucleotide changes.

This study describes the application of HRMA for the rapid detection of key mutations in stored clinical samples from MG infected males and females who attended MSHC during 2007–9. The aim was to determine the prevalence of macrolide resistance mediating mutations (MRMM) in the study population, whether MRMM were contributing significantly to clinical treatment failure, as well and determine the proportion of mutations that were transmitted (present in pre-treatment samples) or selected (present in post-treatment samples only). Azithromycin failure is becoming an increasing clinical problem in the treatment of MG infections, and the application of a rapid molecular assay to identify resistance could facilitate the timely choice of second line agents, which has the potential to reduce transmission of resistant strains and avoid sequelae associated with persistent untreated infection.

## Materials and Methods

### Study samples and controls

Samples were derived from an audit conducted between 11/2007 and 8/2009 at MSHC whereby 111 MG-infected individuals were treated with 1 g of azithromycin and returned for clinical follow-up at one month together with a test of cure (TOC); 86 males and 25 females [16]. Of the 111 patients, stored pre-treatment samples were available from 82 (74%): 20 from women and 62 from men. Of the 26 participants experiencing clinical treatment failure following 1 g of azithromycin in this period, 20 (77%) post-treatment samples were available. Treatment failure was defined microbiologically as a persistently positive PCR approximately 4 weeks after 1 g of azithromycin. A detailed sexual history to indicate risk of reinfection was obtained from participants experiencing treatment failure, but not from those experiencing cure. Positive TOC cases were treated with 400 mg moxifloxacin daily for 10 days, where reinfection was considered unlikely. All samples were either stored at −30°C or stored at room temperature using the DNAstable DNA storage system (Biomatrica, San Diego USA). For the validation of each HRM assay, purified DNA from 18 previously characterized macrolide resistance isolates was used and subsequently used as controls for each 23S rRNA gene mutation type, with *M. genitalium* G37 genomic DNA (ATCC 33530D) serving as the wild type control (Table 1). In addition, 50 consecutive MG negative samples (40 males/10 females) were included to test for nonspecific amplification. Ethics approval, where necessary, was gained from the Alfred Hospital (Melbourne, Australia).

**Table 1:** 23S rRNA gene mutations associated with macrolide resistance in *M. genitalium*.

Mutation (*E. coli* numbering)	Strain	Reference
A2059G	W68551^a^	-
	W1327^a^	-
	W2332^a^	-
	H65213^a^	-
	T63708^a^	-
	M6270	[33]
A2058C	M6302	[17]
A2058G	F61048^a^	-
	F61355^a^	-
	H64377^a^	-
	M16670^a^	-
	W4806^a^	-
	W4564^a^	-
	M6321	[17], [29]
A2058T	F3200^a^	-
	M50367^a^	-
	M14770^a^	-
A2059C	F32379^a^	-
Wild Type	G37	ATCC 33530D

a – Characterized at Statens Serum Institut (Copenhagen, Denmark).

### 23S rRNA gene sequencing

PCR targeting a 147 bp region of the *M. genitalium* 23S rRNA gene was carried out using the primers Mg23S1992F (5′-CCA TCT CTT GAC TGT CTC GGC TAT-3′) and Mg23S2138R (5′-CCT ACC TAT TCT CTA CAT GGT GGT GTT-3′) [17]. The PCR consisted of 10 µl of template DNA, 1× Green GoTaq® Flexi Buffer, 2 mM MgCl_2_ (Promega), 0.2 mM of each dNTP (Invitrogen), 1.25 units of GoTaq® DNA polymerase, and 0.5 µM of each primer PCR in a 50 µl reaction volume. Amplification was performed using a GeneAmp® PCR System 9700 (Applied Biosystems) with the following parameters: activation of the enzyme at 95°C for 10 min, followed by 50 cycles consisting of 95°C for 15 seconds and 60°C for a minute. PCR products were visualized on a 2% agarose gel stained with GelRed (Biotium, Hayward CA USA). PCR amplicons were purified using the Agencourt AMPure XP System (Beckman Coulter, Pasadena CA USA) and quantified using the Qubit® dsDNA HS Assay Kit (Invitrogen, Carlsbad CA USA). DNA sequencing was carried out at the Australian Genome Research Facility using an AB 3730xl capillary sequencer (Applied Biosystems, Foster City CA USA). Sequence analysis was carried out using DNAStar (Lasergene) and SeaView [26]. Accession numbers for each 23S rRNA gene sequence type are shown in Table 1.

### HRMA analysis

Two assays were developed for the discrimination of *M. genitalium* 23S rRNA gene resistance types, by HRMA. Each assay was run on a LightCycler 480 real-time instrument (Roche Diagnostics, Indianapolis USA) with 5 µl template DNA in a 20 µl reaction. The enzyme mix used in this study was the MeltDoc HRM Mix (Applied Biosystems, Foster City USA). Each reaction consisted of an initial PCR amplification step with the same cycling conditions as for the 23S rRNA gene PCR, followed by a 95°C step for one minute, 40°C for one minute, and a melt cycle from 65–95°C at a ramp rate of 0.02°C per second and 25 fluorescent acquisitions per second. The first assay adopted the Mg23S1992F and Mg23S2138R primers used for the 23S rRNA gene sequencing, with the addition of an unlabelled probe phosphorylated at the 3 prime end, Mg23S2053Pb (5′-GAC GGA AAG ACC CCG TGA AG Phos-3′), at concentrations of 40 nM, 200 nM and 200 nM per reaction respectively. The probe implemented in this study shares a 100% homology to the *M. genitalium* 23S rRNA gene wild type sequence. The second assay targeted a 67 bp region of the 23S rRNA gene using the forward primer Mg23S1998F (5′-AAT CCA GGT ACG GGT GAA GA-3′) and the reverse primer Mg23S2080R (5′-CAG TAA AGC TTC ACG GGG TCT-3′) at a concentration of 200 nM per reaction. Oligonucleotide sequences not previously published were designed using Primer3 [27] and the BLAST search engine at the National Center for Biotechnology Information (http://www.ncbi.nlm.nih.gov/BLAST).

### Statistical methods

Data were analysed using PASW software (version 18). Differences between categorical variables were compared using the Chi-square or Fisher's Exact test where appropriate, and proportions were compared using a t-test. Sensitivity and specificity calculations were carried out using DAG_Stat [28].

## Results

### Description of the study population

Of the 111 MG-infected patients treated with 1 g azithromycin who attended for TOC, 77 (69%; 95%CI = 60–77%) were cured with 1 g azithromycin and 34 (31%; 95%CI = 23–40%) were not. Treatment failure was not significantly associated with any demographic or behavioral characteristics including gender or sexual partner from outside Australia (p>0.10). Of these 111 cases, 82 (74%) stored pre-treatment samples (62 male; 20 female) and 20 (77%) post-treatment MG positive samples were able to be sourced and were of sufficient quality to be utilized in this study. There were no significant differences in demographic or behavioral characteristics between the 82 individuals included in the analysis of stored samples and the 29 excluded (Table 2). Importantly, the treatment failure rate in the individuals in whom stored samples were available did not differ from those excluded (28%, 95% = 14–46%), or the overall study population (31%, 95% = 23–40%) (Table 3). The test of cure for MG in the 20 cases included in this study was undertaken at a median of 30 days (range = 14–127 days; IQR = 24.25 days) after treatment. Sexual history was available in 14 of 20 cases of treatment failure: 6 had a probable/definite risk of reinfection, 8 had no risk of reinfection as they were celibate and 6 had an unknown risk with insufficient sexual history available. A risk of reinfection was defined as one being sexually active with a partner between treatment and test of cure. All cases of persistent MG infection following azithromycin were ultimately cured with moxifloxacin 400 mg daily for 10 days.

**Table 2:** Characteristics of patients treated with 1 g azithromycin for MG and presented for a test of cure at Melbourne Sexual Health Centre between December 2007 and December 2009.

	Whole study population (n = 111)	Participants in study (n = 82)	Non-participants (n = 29)	P value^a^
**Mean age +/−SD**	31.3+/−8.4	31.3+/−9.0	31.4+/−6.6	
**Male (%)**	86 (78)	62 (76)	24 (83)	0.43
**Female (%)**	25 (22)	20 (24)	5 (17)	
**Mean number of male partners previous 3 months** b	1.4+/−2.7	1.4+/−2.6	1.4+/−3.0	
**Overseas partner**				
No (%)	77 (70)	57 (70)	20 (69)	
Yes (%)	34 (30)	26 (30)	9 (31)	0.62
**IDU**				
No (%)	107 (97)	79 (98)	28 (98)	
Yes (%)	3 (3)	2 (3)	1 (3)	0.78
**Symptomatic**				
No (%)	11 (10)	9 (11)	2 (7)	
Yes (%)	100 (90)	73 (89)	27 (93)	0.53

a – p value calculated comparing participants to those excluded.

b – period before initial test was positive.

**Table 3:** Summary of 2007–2009 MG positive samples available for 23S rRNA gene mutation screening.

	N
**Total number of MG positive cases (2007–9)**	**111**
Successfully treated with 1 g azithromycin	77 (69%)
Test of cure positive after 1 g azithromycin	34 (31%)
Median time for test of cure testing	35 d (range = 14–127 d)
**Total number cases available for 23S rRNA SNP screening**	**82/111 (74%)**
Successfully treated with 1 g azithromycin	56 (68%)^a^
Test of cure positive after 1 g azithromycin	26 (32%)^b^
Median time for test of cure testing	30 d (range = 14–127 d)

a – 39 male, 17 female.

b – 23 male, 3 female. Of these 26 cases, 20 (18 male, 2 female) corresponding test of cure samples samples were available for analyses. One of these was a persistent case comprising 3 MG positive test of cure samples at 37, 5 and 37 days between positive results.

### Macrolide resistance in pre-treatment *M. genitalium* positive samples

Overall, 23S rRNA gene sequences spanning positions 2058 and 2059 (*E. coli* numbering) were obtained for the 82 pre-treatment MG samples in this study; 16 (19.5%) possessed 23S rRNA mutations (A2058G, A2059G, A2059C) indicating transmitted or circulating macrolide resistance (Information S1). Of the 26 individuals with treatment failure at TOC, 12 (46%) were found to have pre-existing resistance, whereas only 4 (7%) of the 56 individuals with a negative TOC had pre-existing resistance (p<0.0001) (Table 4). Thus, pre-existing resistance was strongly associated with treatment failure following 1 g of azithromycin.

**Table 4:** 23S rRNA gene mutations present in a subset of 2007–2009 MG positive patients (n = 82) with test of cure results available post treatment with 1 g azithromycin.

Sequence type	MG positive after treatment n = 26 (%)	MG negative after treatment n = 56 (%)	P value
**Detection of any 23S rRNA gene mutation**	12 (44)	4 (7)	<0.0001
A2059G	7	3	0.01
A2058G	4	1	0.04
A2059C	1	-	-
**Wild Type**	14	52	<0.0001

### Macrolide resistance in test of cure *M. genitalium* positive samples

The 20 post-treatment samples from individuals with treatment failure after 1 g azithromycin were tested for macrolide resistance mutations. All 20 cases were shown to possess 23S rRNA gene mutations in post-treatment samples (Table 5), consisting of A2059G (60%), A2058G (35%) and A2059C (5%). In 9 (45%) of these cases, mutational changes of the same type were detected in both the pre and post-treatment samples indicating transmitted resistance. Sexual histories on the 5 of 9 cases were available and all but one had been celibate in between treatment and test of cure whilst one had a possible risk of reinfection having been sexually active. Possession of a pre-existing macrolide resistant MG infection was not associated with having an overseas sexual partner. Eleven (55%) cases had a wild type 23S rRNA gene sequence on their pre-treatment sample, but had macrolide mutations detected on post-treatment samples; sexual history was available on 9 of the 11 patients with 4 being celibate in between treatment and TOC, 4 having a possible risk of reinfection having been sexually active and one having a definite exposure to an untreated sexual partner. There was no significant difference observed between the mutation types and whether the patient possessed a pre-existing or selected resistant MG strain. One sample analyzed from a male who originally carried a wild type sequence, possessed both wild type and A2058G sequence types in his positive test of cure sample. It is likely that this patient was tested during the transition phase, as his TOC was carried out 17 days post treatment and there was no risk of re-infection as he was consistently celibate over the period of follow up and TOC.

**Table 5:** 23S rRNA gene mutations present in a subset of 2007–2009 MG positive test of cure cases positive after treatment with azithromycin (n = 20).

Pre-treatment MG sequence type	N	Corresponding post-treatment MG sequence type	N
Wild Type	7	A2059G	7
Wild Type	4	A2058G	4^a^
A2059G	5	A2059G	5
A2058G	3	A2058G	3
A2059C	1	A2059C	1

a – one sample also carried a wild type sequence.

### Macrolide resistance in persistent *M. genitalium* positive case

One of the aforementioned azithromycin treatment failure cases was a male with a positive TOC 37 days after treatment that was confirmed by repeat MG testing. The patient was re-treated with 1 g azithromycin and returned for a second TOC 42 days later that remained positive. This individual had not been sexually-active since initial diagnosis and treatment. At the second positive TOC he was given moxifloxacin 400 mg daily for 10 days and successfully cleared his persistent MG infection. DNA sequencing revealed this patient initially carried a wild type MG infection and that at the first and second TOC only a A2058G resistant type was detected. As this patient demonstrated no reinfection risk it appears that exposure to 1 g azithromycin is likely to have selected for resistance.

### HRMA limit of detection

**Figure 1:**
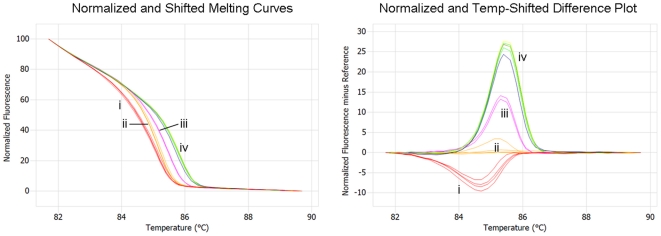
Melt curves and difference plot for MG 147 bp w/unlabelled probe HRMA. i. 23S rRNA gene mutation A2058T (red). ii. Wild type (orange). iii. 23S rRNA gene mutation A2059G (pink). iv. 23S rRNA gene mutations A2058G (blue), A2058C (green) and A2059C (yellow).

**Figure 2:**
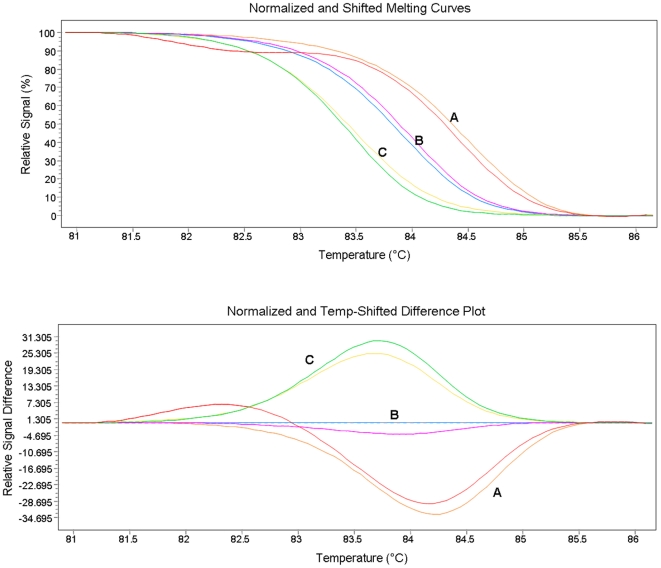
Melt curves and difference plot for MG 67 bp amplicon HRMA. A: 23S rRNA gene mutations A2058C (green) and A2059C (yellow). B: 23S rRNA gene mutations A2058G (blue) and A2059G (pink). C: 23S rRNA gene mutation A2058T (red) and Wild type (orange).

A HRMA assay using a 147 bp amplicon flanking the 23S rRNA gene mutation sites was initially adopted, although an incorporation of an unlabelled probe was necessary to detect the A2058T sequence type. This assay was able to amplify purified *M. genitalium* culture DNA sufficiently to determine this resistance type down to 10–100 copies per reaction, and was able to differentiate the wild type from each mutant types tested (Figure 1), with an average Tm of 85.12°C (SD = 0.27°C). Using a smaller amplicon size of 67 bp (with no unlabelled probe), the limit of detection was able to be improved to ≤10 copies per reaction (average Tm = 83.54°C; SD = 0.33°C); however the wild type and mutant A2058T were difficult to differentiate, as were mutants A2058G and A2059G (Figure 2). Comparable results for both assays were also given using the AccuMelt HRM SuperMix (Quanta Biosciences, Gaithersburg MD USA) and/or use of the Eco Real-Time PCR System (Illumina, San Diego USA).

### HRMA screening of MG positive clinical samples

The 104 MG samples with known 23S rRNA gene sequences (all pre- and post-treatment samples in this study) were screened using both HRMA assays (Table 6). The 147 bp unlabelled probe assay was able to accurately define 94/104 samples (sensitivity = 0.90; 95% CI = 0.83–0.95), with 61/66 (92.4%) samples being wild type, 33/38 (86.8%) resistance types A2058G, A2059G and A2059C. Each of the mutant controls including A2058T were easily discriminated from the wild type samples in this screen. The ten samples that were unable to be classified did not amplify sufficiently to give a useable melt curve, but possessed a 100% homology in 23S rRNA gene sequence to the primers and unlabelled probe used (data not shown). Using the 67 bp amplicon PCR, all 104 samples were able to be accurately defined (wild type versus mutant), although the A2058T mutant controls also grouped with the wild type clinical samples. Upon screening 50 MG negative clinical samples, the 147 bp unlabelled probe assay did not give rise to any nonspecific amplification. The 67 bp assay did give rise to amplification in 12 samples; however the melt profiles were distinguishable with a different average Tm of 82.28°C. In addition, the aforementioned test of cure sample that possessed both wild type and an A2058G mutation showed two clear Tm peaks indicative of a heterozygous sample with both assays.

**Table 6:** HRMA screening of the 23S rRNA gene mutations present in a subset of 2007–2009 MG positive samples (n = 154).

Sequence type	147 bp unlabelled probe assay	67 bp assay
**Detection of any 23S rRNA gene mutation present in the sample set from Melbourne**	33/38 (86.8%)	38/38 (100%)
**Wild Type**	61/66 (92.4%)	66/66 (100%)
**MG negative samples**	0/50 (0%)	0/50 (0%)^a^

a – 12 samples gave rise to amplification of unknown melt profile.

## Discussion

MRMM were detected in all of the azithromycin failure cases investigated from available MG samples collected from an Australian sexual health clinic, between December 2007 and August 2009. We found these treatment failures were due to a combination of both selected resistance during treatment with 1 g azithromycin, and pre-existing transmitted resistant strains, comprising 55% and 45% of the treatment failures, respectively. Interestingly, there was no association with detection of pre-existing resistance and a sexual history of an overseas source. Data from this patient population has shown a treatment failure rate of 31% during 2007–9, an increase from the 16% treatment failure rate previously reported from March 2005–November 2007 [15]. From these data it appears that macrolide resistance is responsible for the azithromycin-treatment failures in this clinical population and that both transmitted and selected resistance is contributing to the observed reduced efficacy of azithromycin.

We have shown that detection of MRMM using rapid molecular methodology such as HRMA is possible on pre and post-treatment samples and that the detection of resistance is significantly associated with treatment failure with 1 g azithromycin. This technology has the capacity, in those with transmitted resistance, to reduce the time to delivery of effective second line agents such as moxifloxacin [15], [29], rather than waiting for a test of cure 2–4 weeks after treatment. It is also able to detect selected resistance in individuals experiencing treatment failure and inform clinicians that a resistant strain rather than reinfection is likely to be responsible for treatment failure. The potential public health benefits of this approach include reducing ongoing transmission of resistant MG strains in the community, and by shortening time to effective second line treatment; it may reduce the incidence of complications such as pelvic inflammatory disease.

While the detection of MRMM was shown to be highly significantly associated with treatment failure, 7% of cases who responded clinically to azithromycin had such mutations detected. This is due to either spontaneous clearance or MG being present below the detection threshold of the diagnostic assays used. The latter explanation is supported by the frequent finding of DNA loads close to the limit of detection in a substantial proportion of patients [10], [30]. A similar finding has been reported in a study by Yew *et al.* where clearance of resistant MG was noted after an extended course of azithromycin, although only in two patients [23]. These patients have not represented with recurrent symptoms at MSHC since having a negative TOC. Furthermore, there is a possibility that patients experiencing treatment failure were re-infected with a different MG strain harbouring the same 23S rRNA gene type, and future studies should consider incorporating genotyping techniques to delineate this possibility [31].

The HRMA assays described in this paper were shown to be effective in the detection of MG 23S rRNA gene mutations associated with macrolide resistance, and were even able to detect a case in which mixed wild type and mutant were present. DNA sequencing, despite being accurate in detecting these nucleotide changes, is not practical for rapid diagnostics. HRMA is an alternative method that is rapid and more cost effective; however, it is recommended by manufacturers of HRMA reagents that a minimum starting template of ≥1.0×10^4^ copies of DNA be used. Consequently, it is not surprising then that the 147 bp amplicon HRMA assay was not able to classify all of the sequenced samples, as a previous study found the average concentration of MG in clinical samples to be 3.0×10^3^ copies per reaction (SD = 5.5×10^3^) [10]. We found that HRMA is indeed capable of characterizing low copy templates, as the use of a smaller amplicon size of 67 bp was able to sufficiently amplify all sequenced samples. This would ordinarily make it the ideal target for an MG HRMA assay; however this assay was unable to detect a type 4 SNP (A2058T). Adenine and thymine residues are accepted to be most difficult to differentiate in DNA melt studies based on a <0.2°C difference in melt temperatures. Attempts were made at incorporating an unlabelled probe to the 67 bp amplicon, but suffered sensitivity issues (data not shown).

Both the 67 bp and 147 bp unlabelled probe HRMA assays are capable of detecting the 23S rRNA types identified in the literature to date relating to clinical resistance from Japan, New Zealand and Scandinavia (A2059G, A2058G and A2058C) [17], [21], [22], [23]: however routine screening carried out at the Statens Serum Institut (Denmark) has identified additional types including those possessing type 4 SNPs (Table 1). The authors consider that specificity outweighs sensitivity in the detection of resistant MG infections and therefore recommend that routine screening of MG positive clinical samples be carried out using the 147 bp unlabelled probe assay, as it will differentiate wild type from 23S rRNA gene mutant in all cases, with the 67 bp assay to be used in cases of low copy number templates. Continued surveillance of the mutation types such as type 4 SNPs is recommended to assess whether they play a significant role with MG macrolide resistance.

The efficacy of azithromycin in published studies to date ranges from 74–96%, with our recent data indicating an efficacy in Melbourne at the lower end of this spectrum. Current recommendations for gonorrhea state that an antibiotic is changed when treatment failure exceeds 5% [32] and this recommendation has also been expanded to cover chlamydia.

The key barrier to provision of highly effective therapy for MG is that the majority of MG infections globally are treated presumptively at the point of diagnosing the STI syndromes of non-gonococcal urethritis and/or cervicitis, and in many settings MG testing for diagnosis and test of cure is not available. Single dose azithromycin has proven to be a highly acceptable and effective for treating these STI syndromes with MG being responsible for only 10–20% of these clinical syndromes in most populations. Single dose (1 g) azithromycin appears to be effective in at least 70% of cases of MG infection while the alternative agent, doxycycline is clearly not sufficiently effective for MG. While there are mixed data, overall extended doses of azithromycin have not proven to be consistently more effective for MG than 1 g doses and are unlikely to be more effective in settings where there is a high level of pre-existing macrolide resistance.

Our interim pragmatic recommendations for management of NGU/cervicitis and MG infections are as follows: cases with urethritis and cervicitis (not PID) are treated with 1 g of azithromycin and tested where possible for MG. Where MG is identified, a TOC is undertaken a month after treatment, and where resistance testing is available this is performed on pre and post-treatment samples to identify the presence and selection of macrolide resistance. We recommend the use of moxifloxacin 400 mg daily for 7–10 in the following situations: i) persistent MG is detected a month following treatment with 1 g of azithromycin without a risk of reinfection, ii) macrolide resistance mutations detected in pre- or post-treatment samples, iii) persistent symptoms of urethritis are present following azithromycin (trichomoniasis is not present) and there is no access to MG testing. We have described in this paper a methodology capable of identifying those patients with MG infection who may not respond to 1 g of azithromycin using a simple, practical and affordable diagnostic assay.

## Supporting Information

Information S1 **FASTA file of MG 23S rRNA sequence types.** File contains the DNA sequence region for the MG wild type and macrolide resistance mutations A2059G, A2058G, A2059G, A2058C and A2058T.
(TXT)
